# Sensorial function in children with congenital Zika syndrome: what is the relationship with motor function?

**DOI:** 10.1590/1984-0462/2026/44/2025112

**Published:** 2026-01-19

**Authors:** Ana Stela Salvino de Brito, Chanazy Ayalla de Castro Meira, Karinny Michelle Alves Moreira, Thayla Amorim Santino, Jousilene de Sales Tavares, Gabriela Lopes Gama, Adriana Melo

**Affiliations:** a Instituto Assistencial Professor Joaquim Amorim Neto, Campina Grande, PB, Brazil. Instituto Assistencial Professor Joaquim Amorim Neto Campina Grande PB Brasil; b Universidade Estadual da Paraíba, Campina Grande, PB, Brazil. Universidade Estadual da Paraíba Campina Grande PB Brasil; c Universidade Federal de Juiz de Fora, Governador Valadares, MG, Brazil. Universidade Federal de Juiz de Fora Governador Valadares MG Brazil

**Keywords:** Microcephaly, Motor disorders, Perception, Zika virus infection, Microcefalia, Transtornos motores, Percepção, Infecção por Zika vírus

## Abstract

**Objective:**

To investigate sensory impairments in children with congenital Zika syndrome (CZS) and the relationship with motor function.

**Methods:**

This is a cross-sectional, analytical study of medical records of children with CZS who were assisted by a support center for children with microcephaly in northeastern Brazil. General data about the children and their caregivers were recorded. Data from the Gross Motor Function Measure (GMFM-88) score and Gross Motor Function Classification System classification were recorded for motor function assessment, and the Sensory Profile 2 score was recorded for sensory function.

**Results:**

A total of 32 children with a mean age of 53.4±10.8 months participated in the study. The GMFM-88 score was positively correlated with the total score of the Sensory Profile 2 (r=0.49, p=0.004) and with the scores for tactile (r=0.44, p=0.011), vestibular (r=0.41, p=0.019), and proprioceptive (r=0.35, p=0.045) systems. When adjusted for age, only children under 50 months old (n=16) presented these correlations (tactile: r=0.69, p=0.003, vestibular: r=0.74, p=0.001, and proprioceptive: r=0.63, p=0.009).

**Conclusions:**

The tactile, vestibular, and proprioceptive systems were correlated with the gross motor function in children under 50 months old with CZS. These findings suggest the clinical relevance of considering both sensory and motor domains in the evaluation and care of children with CZS, supporting the need for early and integrated sensory-motor interventions.

## RESUMO

**Objetivo:**

Este estudo teve como objetivo investigar as alterações sensoriais vivenciadas por crianças com síndrome congênita do Zika (SCZ) e sua relação com a função motora.

**Métodos:**

Trata-se de um estudo transversal, retrospectivo, quantitativo e analítico, cujos dados foram coletados de prontuários de crianças com SCZ atendidas em um centro de apoio à criança com microcefalia localizado no nordeste do Brasil. Foram registrados dados gerais sobre as crianças e seus cuidadores. Os dados da pontuação da Medida da Função Motora Grossa (GMFM-88) e da classificação do Sistema de Classificação da Função Motora Grossa foram registrados para avaliação da função motora, e a pontuação do Perfil Sensorial 2 foi registrada para a função sensorial.

**Resultados:**

Participaram do estudo 32 crianças com idade média de 53,4±10,8 meses. A pontuação do GMFM-88 correlacionou-se positivamente com a pontuação total do Perfil Sensorial 2 (r=0,49, p=0,004) e com as pontuações dessa escala referentes aos sistemas tátil (r=0,44, p=0,011), vestibular (r=0,41, p=0,019) e proprioceptivo (r=0,35, p=0,045). Quando ajustado pela idade, apenas crianças menores de 50 meses (n=16) apresentaram essas correlações (tátil: r=0.69, p=0.003, vestibular: r=0.74, p=0.001 e proprioceptivo: r=0.63, p=0.009).

**Conclusões:**

Os sistemas tátil, vestibular e proprioceptivo apresentaram correlação com a função motora grossa de crianças com SCZ e menores de 50 meses. Esses achados sugerem a relevância clínica de considerar conjuntamente os domínios sensorial e motor na avaliação e no cuidado de crianças com SCZ, reforçando a necessidade de intervenções sensório-motoras precoces e integradas.

## INTRODUCTION

In 2015, the Zika virus was first detected in Brazil, causing an epidemic outbreak recognized worldwide for its severe effects on fetal development.^1^ In 2016, the relationship between intrauterine Zika virus infection and fetal brain abnormalities was confirmed,^2^ characterizing congenital Zika syndrome (CZS). Since 2015, some clinical signs of CZS have been described as microcephaly; growth restriction; changes in muscle tone; visual, auditory, and cognitive alterations; difficult-to-control seizures and severe motor impairments.^3,4,5,6,7,8,9^

Currently (a decade after the first described cases), the severe impairments in children with CZS still draw attention.^10^ These children generally have severe motor impairments resulting in restricted prone and supine postures and difficulty in sitting without support due to the absence of protective reactions and balance.^11^ Impairments in motor function have been associated with comorbidities such as epilepsy and dysphagia.^8^ In addition, sensory impairments in visual, auditory, vestibular, proprioceptive, and tactile systems have been reported.^5,6,12,13,14,15^ Despite these findings, to the best of our knowledge, the relationship between sensory impairments and motor function has not yet been thoroughly investigated in a sample composed exclusively of children with CZS.

In this context, this study aimed to describe sensory impairments in children with CZS and assess the relationship between sensory and motor functions. A better understanding of the sensory impairments in children with CZS and their relationship with motor function may guide multidisciplinary therapeutic approaches to minimize sequelae and help recognize the systemic consequences of brain impairments.

## METHOD

This is a cross-sectional, quantitative, and analytical study of medical records from a support center for children with microcephaly in Campina Grande, Paraíba, Brazil. This study was approved by the research ethics committee of Centro de Ensino Superior e Desenvolvimento (CESED) (no. 5.379.175, Certificate of Presentation for Ethical Consideration — CAAE: 58068922.1.0000.5175) and followed the Declaration of Helsinki. Data collection was carried out between May 2022 and August 2022, and those responsible for the institution authorized the access to the medical records before data collection.

This study included medical records of children diagnosed with CZS, confirmed by reverse transcriptase polymerase chain reaction (RT-PCR) or imaging tests (e.g., computed tomography and magnetic resonance), performed in the first months of life, and who had been evaluated for sensory and motor function at least once. The imaging examinations were interpreted by a physician specialized in fetal medicine, with recognized expertise in evaluating neuroimaging findings associated with congenital Zika virus infection. Children with incomplete medical records, severe sensory alterations (e.g., complete blindness or deafness), and other developmental disabilities were excluded. Children for whom sensory and motor assessments were performed at an interval longer than one week were also excluded. These criteria were defined to reduce the risk of significant changes in the child’s clinical condition between assessments, which could compromise the evaluation of the relationship between motor and sensory function.

Children’s general data (e.g., sex, weight, head circumference [HC], and length at birth), gestational age at birth, and type of delivery were collected from medical records, as well as motor and sensory assessments.

Retrospective data from the Gross Motor Function Measure (GMFM-88) and Gross Motor Function Classification System (GMFCS) were used to assess the motor function. GMFCS is an ordinal classification widely used to assess the ability to move and functionality based on age, ranging from level I (walks without limitations) to V (transported in a wheel-chair).^16,17^ The GMFM-88 assesses the ability to perform static and dynamic motor tasks without help. It has 88 items grouped into dimensions: lying down and rolling (A); sitting (B); crawling and kneeling (C); standing (D); and walking, running, and jumping (E). Each task must be scored between 0 (cannot do) and 3 (task completion), totaling a maximum final score of 264 points.^18^

Sensory function was assessed using retrospective data from the Sensory Profile 2.^19^ This instrument evaluates sensory function for possible changes during daily activities according to caregivers. This study considered only the results of the auditory, visual, tactile, vestibular, oral, and proprioceptive systems. In each item, the caregiver should indicate how often the child showed a certain response in different situations: almost always,^5^ often,^4^ half the time,^3^ occasionally,^2^ or almost never.^1^ The sum results in a raw score; higher scores indicate a greater frequency of sensorial response. Based on the raw score, these responses can be classified using the expected results of the normal curve: much less than expected, much less than others (-2 standard deviations [SD]); less than others (-1 SD); just like the majority of others, more than others (+1 SD); and much more than others (+2 SD).^20^

All assessments were performed by trained physiotherapists and occupational therapists who work at the support center where the study was conducted, as part of the institution’s assessment protocol. These professionals are specialized in caring for children with neurological impairment and have extensive experience in caring for children with CZS.

The Statistical Package for the Social Sciences (SPSS) software, version 23.0 (IBM Corp., USA), was used for data analysis. Descriptive statistics were performed using measures of central tendency and dispersion for continuous variables (e.g., age, weight, length, and HC at birth) and relative and absolute frequencies for city of residence, microcephaly at birth, and other common imaging findings. Pearson’s correlation coefficient analyzed the GMFM-88 with the score for each sensory system, considering all children and subgroups based on age (> 50 months [n = 16] and < 50 months [n = 16]). The 50-month cutoff was selected to ensure comparable group sizes and to enhance the interpretability and reliability of the comparative analysis.

## RESULTS

A total of 32 children (12 females) aged between 35 and 80 months (mean 53.4±10.8) at the sensory and motor assessments were included in the study. Of these, 50.0% were over 50 months, 46.9% had severe microcephaly at birth, and 90.6% were classified as level V, according to the GMFCS. Table 1 shows the general characteristics assessed during pregnancy and postpartum.

**Table 1:** General characteristics of children with congenital Zika syndrome during pregnancy and postpartum.

Variables	n (%)	Mean±SD	Range
Sex			
Female	12 (37.5)		
Male	20 (62.5)		
Skin rash during pregnancy			
No	5 (15.6)		
Yes	27 (84.4)		
GA (weeks) of skin rash		13±7.1	6–32
Duration of skin rash (days)		3.9±2.8	1–15
Trimester of occurrence of skin rash			
First	16 (50.0)		
Second	9 (28.1)		
Third	2 (6.3)		
GA at birth (weeks)		38.7±1.6	35–41
Type of delivery^*^			
Cesarean section	9 (28.1)		
Vaginal	22 (68.8)		
Prematurity^*^			
No	27 (84.4)		
Yes	4 (12.5)		
Birth weight (g)		2771.8±473.1	1750–3555
Birth length (cm)		45.6±3.4	36–50
Birth HC (cm)		29.8±1.7	26–33
Birth microcephaly^*^,^†^			
No	6 (18.8)		
Mild	9 (28.1)		
Severe	15 (46.9)		
Arthrogryposis^*^			
No	31 (96.8)		
Yes	0		
Current age (months)			
>50	16 (50.0)		
<50	16 (50.0)		
Current weight (g)		14814.1±3872.7	10,430–27,610
Current length (cm)		98.4±9.0	83.5–121
Current HC (cm)		42.5±3.3	37.3 – 52
GMFCS			
Level II	1 (3.1)		
Level IV	2 (6.3)		
Level V	29 (90.6)		

SD: standard deviation; GA: gestational age; HC: head circumference; GMFCS: Gross Motor Function Classification System.

^*^One child was adopted and did not have some birth data;

^†^One child had incomplete birth data.

In the GMFM-88 assessment, only three children scored on tasks from dimension C and two children on dimensions D and E. The total GMFM-88 scores ranged from 7 to 223 points, with a mean of 30.56±41.4 points. In the Sensory Profile 2, visual and tactile systems were the most frequently compromised, followed by vestibular and proprioceptive systems (Table 2). The total Sensory Profile 2 score ranged from 27 to 140 points, with a mean of 88.1±22.7 points. Table 3 shows the scores for GMFM-88 by dimension and Sensory Profile 2 by system.

**Table 2:** Characterization of children with congenital Zika syndrome according to the normative result of Sensory Profile 2.

	Sensory Profile 2 classification, n (%)				
Sensory system	Much less than most children	Less than most children	Just like the most children	More than most children	Much more than most children
Auditory	0	1 (3.1)	25 (78.1)	6 (18.8)	0
Visual	1 (3.1)	10 (31.3)	19 (59.4)	2 (6.3)	0
Tactile	0	7 (21.9)	19 (59.4)	5 (15.6)	1 (3.1)
Vestibular	0	8 (25.0)	22 (68.8)	1 (3.1)	1 (3.1)
Oral	0	4 (12.5)	27 (84.4)	1 (3.1)	0
Proprioceptive	0	6 (18.8)	22 (68.8)	3 (9.4)	1 (3.1)

**Table 3:** Scores in the Sensory Profile 2 and Gross Motor Function Measure-88.

	Mean±SD	Range
GMFM-88		
Dimension A	16.69±11.78	13.00 (5–51)
Dimension B	9.41±6.50	6.50 (0–60)
Dimension C	2.00±7.80	0.00 (0–39)
Dimension D	0.97±5.30	0.00 (0–30)
Dimension E	0.00±7.62	1.50 (0–43)
Sensory Profile 2		
Auditory system	19.53±5.24	19.00 (9–30)
Visual system	10.44±4.41	10.00 (5–21)
Tactile system	13.47±7.34	11.00 (5–33)
Vestibular system	11.53±6.60	11.50 (2–31)
Oral system	13.78±7.19	16.00 (0–23)
Proprioceptive system	12.38±5.68	12.50 (1–28)

SD: standard deviation; GMFM-88: Gross Motor Function Measure.

**Figure 1:**
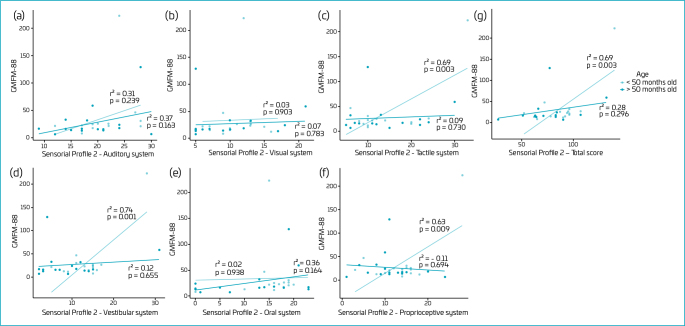
Correlation between Gross Motor Function Measure (GMFM-88) and Sensory Profile 2 according to age. (a) Auditory system; (b) Visual system; (c) Tactile system; (d) Vestibular system; (e) Oral system; (f) Proprioceptive system; (g) Total score.

The total GMFM-88 score was significantly correlated with Sensory Profile 2 scores (r=0.49, p=0.004) and scores for tactile (r=0.44, p=0.011), vestibular (r=0.41, p=0.019), and proprioceptive (r=0.35, p=0.045) systems. When adjusted for age, significant correlations were found for the same systems only for children under 50 months (n=16) (Figure 1).

## DISCUSSION

The results presented in this manuscript provide more information about sensory impairments in children with CZS aged over 34 months. In addition, this study demonstrates the relationship between motor and sensory impairments in children under 50 months old with CZS, particularly between motor function and tactile, vestibular, and proprioceptive systems. Severe impairments in motor function in children with CZS have been widely described in the literature, and previous studies showed that most children cannot perform tasks in dimensions C, D, and E of GMFM-88,^8,9,21,22,23^ corroborating our results. Additionally, some studies have described changes in auditory and visual systems, with the visual system being the most reported in the literature,^5,24,25,26,27^ possibly due to the high prevalence of impairments in this system, which was also observed in the present study.

Carvalho et al. evaluated the sensory function of 17 children with microcephaly, most of whom (n=12) were diagnosed with CZS. Unlike our results, these authors reported that most children presented impairments in auditory, tactile, vestibular, and oral systems. Several factors may account for these discrepancies, including potential differences in the diagnostic criteria applied to the evaluated children and in the extent of neurological impairments, as well as variations in age, neuropsychomotor development, and sensory experiences among the children. On the other hand, most children evaluated in this study presented typical performance in the visual system, which the authors attributed to the difficulty parents may have in identifying visual impairments in young children with neurological and psychomotor developmental delays.^28^ In the present study, 40.6% of the evaluated children presented impairments in the visual system. This finding may be explained by children’s age, level of development, and a potentially stronger parent-child relationship, which may facilitate the recognition of visual impairments.

Despite the sensory and motor impairments described in children with CZS, their relationship still needed to be elucidated. This relationship was already described in children with cerebral palsy.^29,30,31,32^ In general, the sensory system represents an important source of information to correct motor actions and is essential for motor performance,^33^ and children with cerebral palsy classified in levels IV and V of the GMFCS have greater sensory impairment.^30^ In children with CZS, this relationship may be explained by the brain abnormalities from the infection,^34^ which may compromise organization, integration, synthesis, and use of responses from sensory information to generate adaptive responses to the environment.

This study confirmed the relationship between both functions and highlighted two key findings: first, the relationship of motor function only with the tactile, vestibular, and proprioceptive systems; and second, that this relationship only occurs in children under 50 months old. The first finding may be explained by the motor activities assessed in this study. Although motor function is classified into fine and gross, only gross function was assessed, which is mainly influenced by tactile, vestibular, and proprioceptive information.^30^

In children with neurological impairment, sensory functions may be compromised by changes in sensory processing (due to brain damage), muscle tone, posture, and movement patterns, which may limit motor planning and learning through the years.^30^ Thus, brain and musculoskeletal abnormalities (e.g., joint deformities, muscle shortening, and tone changes) may have influenced the results observed in children over 50 months old. However, further studies need to confirm this hypothesis.

This study is a pioneer in widely investigating sensory function exclusively in children with CZS aged above one year, considering tactile, vestibular, proprioceptive and somatosensory systems, as well as their relationship with motor function. These results highlighted the need for interventions on motor and sensory functions, especially in children under 50 months old, whose sensory function is related to motor function. Cemali et al.^35^ showed that combining sensory integration and physical therapy is more effective than physical therapy alone. In addition, sensory integration positively affected gross motor function (especially in sitting, crawling, and standing) in children with cerebral palsy aged between 24 and 72 months old.^36^

Despite the importance of the results, this study had some limitations, such as the lack of control over the interventions performed by children, the presence of brain and musculoskeletal impairments (e.g., shortening and contractures), and the absence of a control group. In addition, all children had severe motor impairments, most of them classified as level V of the GMFCS, which may limit the generalizability of the results. Another important limitation is related to the study design, since data were collected from clinical records, evaluation bias should be considered. Moreover, the use of Sensory Profile 2 presents an inherent limitation, as this instrument relies on parental responses to evaluate children’s sensory function. However, this tool has been widely used in both clinical practice and research involving children’s sensory function. Thus, future studies should include children classified with other GMFCS levels and investigate the impact of interventions for sensory integration on motor function and consider different age subgroups to verify whether the observed relationship persists across developmental stages.

A relationship of the gross motor function with the tactile, vestibular, and proprioceptive systems was observed in children with CZS under 50 months old but not in those above this age. These findings highlight that sensory and motor functions are associated in early childhood, reinforcing the clinical importance of considering both domains in the evaluation and care of children with CZS, as well as the need for early and integrated sensory-motor interventions. However, these associations do not establish a cause-and-effect relationship. Thus, future studies should explore the relationship between tailored interventions and changes in sensory and motor function and participation of children with CZS.

## Data Availability

The database that originated the article is available with the corresponding author.
